# Thousands of Years of Pastoralism Don’t Count: Coprophagous Beetles Prefer Exotic Alpaca Dung to That of Cattle

**DOI:** 10.3390/insects15120934

**Published:** 2024-11-27

**Authors:** Antonio Rolando, Daniele Bertolino, Alex Laini, Angela Roggero, Claudia Palestrini

**Affiliations:** 1 Dipartimento di Scienze della Vita e Biologia dei Sistemi, Università di Torino, Via Accademia Albertina 13, 10123 Torino, Italy; antonio.rolando@unito.it (A.R.); daniele.bertolino@edu.unito.it (D.B.); angela.roggero@unito.it (A.R.); claudia.palestrini@unito.it (C.P.); 2 National Biodiversity Future Center (NBFC), 90133 Palermo, Italy

**Keywords:** alien species, Scarabaeoidea, insect conservation

## Abstract

Dung beetles mostly feed on mammal dung. Throughout the European Alps, the dung produced by local domestic ungulates attracts many species of dung beetles, giving rise to rich and diversified communities that play an important role in the Alpine agricultural ecosystem. There is, therefore, understandable concern about the introduction of exotic livestock, such as alpacas (*Vicugna pacos* (Linnaeus, 1758)), into the region. This research studied dung beetle assemblages in an Alpine valley where both cattle and alpacas are raised. We used standardized pitfall traps baited with alpaca or cow dung along altitudinal transects to assess the “attractiveness” of the two resources to dung beetles. Most species entered both trap types. The average number of species per trap did not vary significantly and the nMDS trap ordination largely overlapped, but the total number of individuals and the average number of individuals per trap were higher in traps baited with alpaca dung. This difference was largely due to the preference of the dominant species *Euheptaulacus carinatus* (Germar, 1824) for alpaca dung-baited traps. Moreover, both the abundance and specific richness changed with altitude, being greatest in alpaca traps during July at intermediate altitudes. IndVal analyses showed that eight out of nine species (all Aphodiinae) showed a preference for alpaca traps. Since the microclimatic conditions (i.e., temperature) of the two trap types were virtually the same, it is reasonable to consider trophic preferences as the source of the observed differences. Finally, we also sampled the beetles present in the alpaca latrines and cow dung pats deposited on pastures, providing confirmation that dung beetles do indeed visit the dung of both species. Our results emphasize the usefulness of polyphagia and the trophic flexibility of dung beetles, providing evidence that the long history of pastoralism in the Alps has not led local species to adapt to the dung of domestic ungulates. The results also suggest that alpaca breeding could be a sustainable activity since, thanks to the work of dung beetles which feed upon and move the dung into their tunnels (as occurs with cattle dung), there is less need for farmers to remove the dung from pastures.

## Simple Summary

The European Alps host many species of dung beetles that feed on domestic and wild ungulate’s dung and provide many benefits to agricultural ecosystems. However, it is not clear whether dung beetles will feed on the dung of species introduced for commercial purposes from other geographical areas. Here, we investigated dung beetle feeding preferences by providing them with cow (local) and alpaca (introduced) dung in four pastures along an altitudinal gradient. We found that the number of species and abundance in the alpaca dung equals or exceeds those found in the cow dung. Moreover, some species showed clear preferences for alpaca dung while few preferred cow dung. Overall, our results suggest that dung beetle plasticity in feeding habits allows them to readily exploit the dung of introduced species and that alpaca dung can help maintain dung beetle richness in the studied area.

## 1. Introduction

Dung beetles feed on dung, and it has long been held that they primarily feed on dung deposited by mammals. That said, recent studies have suggested that the diet of many dung beetle species may be far more diversified than previously believed. For instance, the results of experiments conducted in a laboratory setting suggest that their use of arthropod carcasses as a food source may have been largely underestimated [1]. DNA analyses of dung beetle gut content also revealed their consumption of rodent dung to be more prevalent than previously thought [2,3]. Although adult dung beetles are generally polyphagous and opportunistic, they also show clear trophic preferences. The use of pitfall traps baited with mammalian feces showed certain species or assemblages to be differentially attracted to distinct dung types [4,5,6,7,8,9,10,11]. The composition of the volatile organic compounds (VOCs) emitted by the dung (which varies according to livestock species and diet) is thought to be the key element responsible for shaping food preferences [10,12].

Although adults and larvae may consume the same dung, evidence obtained for some species suggests that the types of feces adults feed on are different to those used for larval rearing, reflecting a shift in resource use according to life stage [3]. This observation is in keeping with the fact that larvae still have chewing mandibles and may be able to take advantage of the solid parts of dung (grass fibers and other undigested material), while the adults only consume the liquid parts [13,14] or ingest minute particles at most [15].

Dung choice may be relevant to the general health and the reproduction of dung beetles. In *Thorectes lusitanicus* (Jekel, 1865), acorn consumption appeared to confer potential advantages. For example, acorn-fed beetles showed significant improvements in their fat body mass, hemolymph composition, and ovary development. Moreover, during the reproductive period (October–December) beetles incorporating acorns into their diets were more likely to exhibit better resistance to low-temperature conditions [16]. With regard to reproduction, the individuals of *Onthophagus lecontei* Harold, 1871 reared in native wild rabbit dung produced more progeny, more brood masses and larger adult beetles and the offspring remained in each preimaginal stage for a shorter period than those reared on horse or goat dung [17].

Diet preferences may be the result of coevolution with local fauna. It is very indicative, in this regard, that dung beetles developed markedly generalist diets to survive in territories devoid of large mammals in Australia [18] and avian-dominated territories in New Zealand [19,20]. Most native Australian dung beetle species co-evolved with the small, hard, dry, pellet-like dung produced by marsupials in forests and woodland habitats and are not capable of using the large wet cattle dung pads on open grasslands [21].

Throughout the European Alps, dung beetles have long co-occurred (and, possibly, coevolved) with livestock. Mitochondrial DNA studies indicate that the domestication of cows (*Bos taurus* Linnaeus, 1758) started in Southwest Asia in the 9th millennium BC. Domesticated cattle were then introduced into Europe during the Neolithic transition, around 6400 BC [22]. Domesticated sheep (*Ovis* spp.) also dispersed across Europe via several migratory episodes at about the same time [23]. Archeological studies in the Alps have provided definite proof of alpine farming and pasturing in the Bronze Age (2200 to 800 BC). Palynological studies found indicators of high-altitude pasture use even earlier, dating to around 4500 BC [24,25,26]. These data are also in keeping with the vegetational changes that took place during the period of early pastoralism, occurring some 7000 to 5000 years ago [27].

We hypothesized that thousands of years of livestock grazing in the Alps could have triggered a process of adaptation of dung beetles to the dung produced by domestic ungulates, especially cattle and sheep. This adaptation may also have affected their intestinal microbiota. Dung beetles rely on the presence of both obligate and facultative symbionts (bacteria and fungi) in their gut to satisfy many physiological needs [28,29]. Certain microbes can be acquired by dung beetles during the consumption of different types of feces [14,30,31], but others seem to be species-specific, probably resulting from a variety of processes occurring along different time scales, such as parental vertical derivation [32,33] or a species’ evolutionary history [34,35], perhaps mediated by differences in intestinal morphology [33]. This hypothetical adaptation process could be boosted by the fact that the dung of livestock may attract more individuals than the dung of wild herbivores [11].

Research carried out on the pastures of the European Alps indicate that cattle dung attracts a multitude of species, resulting in rich and diversified dung beetle communities [36,37,38,39,40,41] which, in turn, provide important ecosystem services [42]. The introduction of exotic domestic ungulates might pose a threat to this delicate balance. In both the United States and Australia, the native dung beetle fauna present at the time of their introduction were not able to utilize the droppings produced by the livestock. This created the need for other dung beetle species able to degrade the dung, and these new species caused significant changes to the indigenous dung beetle communities [21,43].

The introduction of exotic livestock into the European Alps is, therefore, an issue of understandable concern. Indeed, hundreds of Alpine farmers are now choosing to raise llamas or alpacas to supplement their dwindling incomes. The alpaca (*Vicugna pacos*) is a camelid native to the Andes. In the Alps, it is being bred both for its fleece and as a tourist attraction. In theory, its breeding is a rather sustainable practice. Alpacas tend to defecate (usually in the form of pellet droppings) and urinate in a few concentrated areas (communal latrines), thus ensuring a certain hygiene in the local ecosystems. Since their hooves are padded with soft cushions, their presence is also not associated with any significant damage to the soil or grass. Finally, because they lack teeth in their upper palette, they gently pluck grasses without damaging pastures.

The present research considers the dung beetle communities of an Alpine valley in Italy where both alpacas and cows are grazed. To determine which type of dung was most attractive to the population of local dung beetles, we used standardized pitfall traps baited with alpaca or cow dung along altitudinal transects. Subsequently, we conducted some hand collections in both alpaca latrines and cow dung pats. In this way, we intended to verify that the latrines were indeed used by dung beetles.

Considering the long history of cattle pastoralism in the Alps, the phylogenetic divergence between alpacas (camelid) and cows (bovid), and the intrinsic and extrinsic characteristics of alpaca feces (which are dry, small, and bean-shaped, and thus very distinct from cow pats), we expected that dung beetles would exhibit a preference for cow dung over the recently introduced alpaca dung.

## 2. Materials and Methods

The study was carried out in the years 2023 and 2024. The only feed source for both the alpaca and cow herds was the pasture’s grasses, and no animals received any veterinary treatments.

### 2.1. Pitfall Trapping

**Figure 1:**
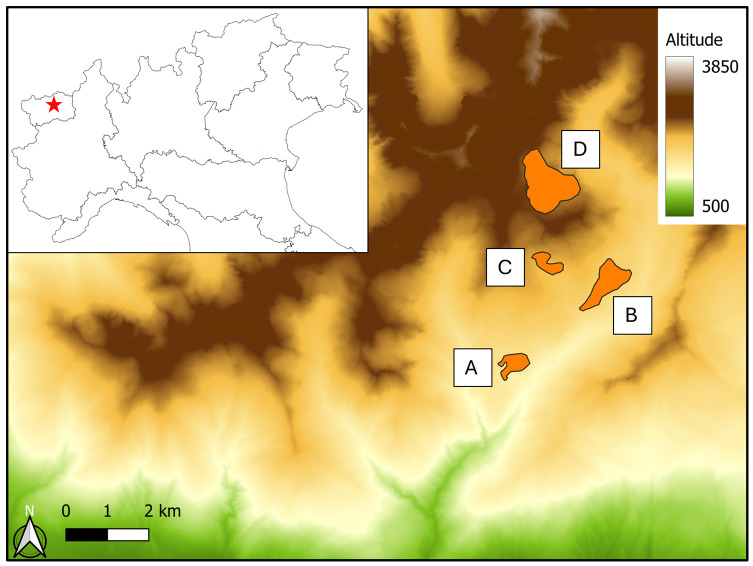
Study area, located in northwestern Italy. Traps for collecting dung beetles were positioned in four pastures (A, B, C, D; highlighted in orange) at different altitudes.

We sampled dung beetles along an altitudinal transect that consists of four south-oriented pastures (pastures A, B, C, and D; Figure 1) located at different altitudes (1650, 1900, 2150, and 2450 m a.s.l., respectively). In each pasture, four groups of three pitfall traps (two groups baited with alpaca dung and two with cow dung; total number of traps = 12 traps) were randomly positioned in each corner of an area measuring approximately 200 m × 200 m. The three traps of each group were placed at the vertices of an equilateral triangle measuring 50 m on each side. Each trap consisted of a 1.5 L clear plastic bottle, 9 cm in diameter, which was cut in half (at about 20 cm from the top). The top half of the bottle provided a funnel, which was then inserted upside-down into the bottom half of the bottle (approx. 25 cm in height). The traps were baited with the same volume (200 mL) of fresh cattle or alpaca dung, collected in the pastures and suspended in a fine mesh plastic bag on a tripod made with three sticks, 50 cm in length, placed over the trap close to the entrance of the funnel. Dung beetles were collected every 10 days, from 6 June to 20 September 2023. Sampling from pasture D started at the end of July due to the persistence of snow cover. A total of 480 traps were thus sampled.

Temperature was recorded every four hours using Thermo-Button miniature data loggers placed at the center of each group of three traps (i.e., four data loggers per site). These small buttons (Ø × H: 16 mm × 6 mm) were attached to a wooden stake driven into the ground to measure the temperature of the air just above the surface of the soil.

### 2.2. Dung Beetle Sampling in Dung Pats and Latrines

The alpacas were kept by farmers at a low altitude in pastures located approximately 600 m from pasture A. We verified the dung beetles’ use of alpaca latrines and cow dung pats in this area in July (on two separate occasions) and August 2024 (on one occasion). When the alpacas started a new latrine, we placed an appropriate number of fresh cow dung pats in the same area. Subsequently, care was taken to sample the beetles present in latrines and pats of the same age (3, 6, 9, and 12 days old) and size (since the cow dung pats were 20 cm in diameter, we sampled an area measuring 20 cm in diameter within the latrine).

### 2.3. Data Analysis

To assess whether differences in temperature between treatments may have influenced the dung beetles’ preference for one pitfall trap over another, we used a linear mixed-effect model (LMM) with site as the random effect and an autoregressive moving-average (ARMA) correlation structure to consider temporal autocorrelation.

We evaluated the effect of dung type on species richness and abundance using generalized linear mixed-effect models (GLMM) with site and date as random effects. The error distribution was set to negative binomial for species richness and generalized Poisson for abundance to cope with over- or under-dispersion. Confidence intervals for the dung type were calculated using the profile method. The effect of dung type was considered significantly different from 0 if the confidence interval did not include 0.

We evaluated community structure via non-metric multidimensional scaling (nMDS), using Bray–Curtis dissimilarity and stress as a measure of the goodness of fit. The effect of dung type was qualitatively evaluated by superimposing convex hulls over the nMDS plot.

Analysis was performed using the packages *nlme* version 3.1.164 [44], *glmmTMB* version 1.1.9 [45] and *vegan* version 2.6.6.1 [46] in R software for statistical computing [47]. Model assumptions of GLMMs were checked using the *DHARMa* package version 0.4.6 [48]. We calculated IndVal [49] with the *indicspecies* package version 1.7.14 [50] to identify the species that were characteristic of alpaca traps and cow traps.

## 3. Results

### 3.1. Pitfall Trapping

#### 3.1.1. Temperature

Temperature did not differ significantly between alpaca vs. cattle treatments (F_1,14_ = 0.132, *p* = 0.722), meaning that the temperature of the traps baited with either cow or alpaca dung was virtually the same in all pastures.

#### 3.1.2. Spatio-Temporal Community Composition

Of the 480 traps that were positioned, 67 were damaged and thus excluded from the calculations. Dung beetle community composition was subject to spatio-temporal conditioning. Certain species were most prevalent in the low-altitude pasture A (*Limarus zenkeri*, *Onthophagus joannae*), in the intermediate pastures B and C (*Euheptaulacus carinatus*), or in the high-altitude pasture D (*Oromus alpinus*) (Table 1). Some species appeared at the beginning of the summer (*Onthophagus joannae*, *Colobopterus erraticus*, *Esymus pusillus*), while others appeared later (*Planolinus fasciatus*).

#### 3.1.3. Species Richness and Species Abundance

A total of 21,675 individuals were collected and 26 species were identified. The Aphodiinae subfamily was the most represented, with 19 species, followed by Scarabaeinae (4 species) and Geotrupinae (3 species). *E. carinatus* was by far the most abundant species, with 17,313 individuals sampled, followed by *Bodilopsis rufa*, with 1246, and *Onthophagus fracticornis*, with 891 (Table 1).

**Figure 2:**
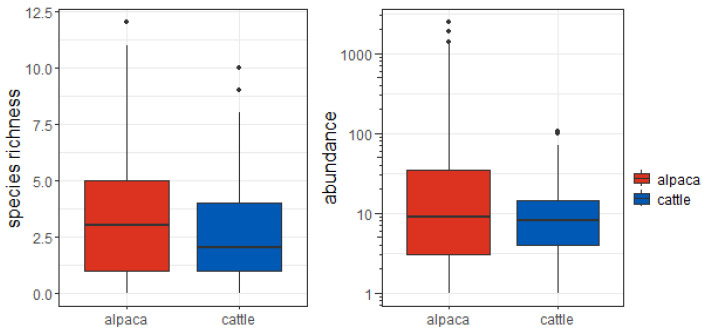
Mean number of species and individuals (abundance) per trap. The abundance is reported on a logarithmic scale with base 10.

Most species were caught in both trap types, with the noticeable exception of *Limarus zenkeri* (all 58 individuals were caught in alpaca traps). Accordingly, there was no significant effect of dung type on species richness (lower C.I. = −0.421; upper C.I. = 0.173) (Figure 2, left-hand side).

Alpaca dung traps captured many more individuals than cow dung traps (19,395 vs. 2280). Accordingly, the effect of dung type on abundance was significant (lower C.I. = −2.79; upper C.I. = −1.59), with alpaca traps attracting more individuals (Figure 2, right-hand side). Although this difference was predominantly due to the 16,984 individuals of *E. carinatus*, the alpaca dung traps hosted more individuals than cow dung traps (2411 vs. 1951) even if this species was excluded from the calculation.

**Figure 3:**
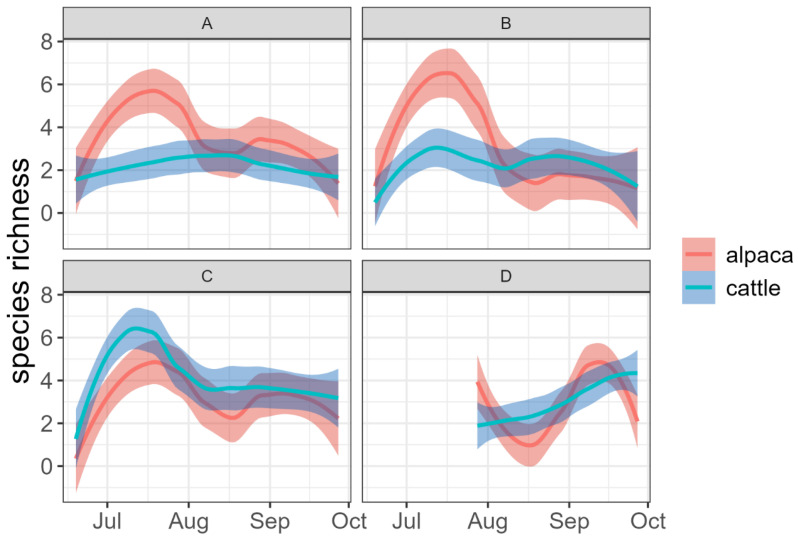
Trends in the number of species (species richness) over time at the four pastures (A, B, C and D). In the high-altitude pasture (D), sampling began later than in the others due to the persistence of snow cover.

**Figure 4:**
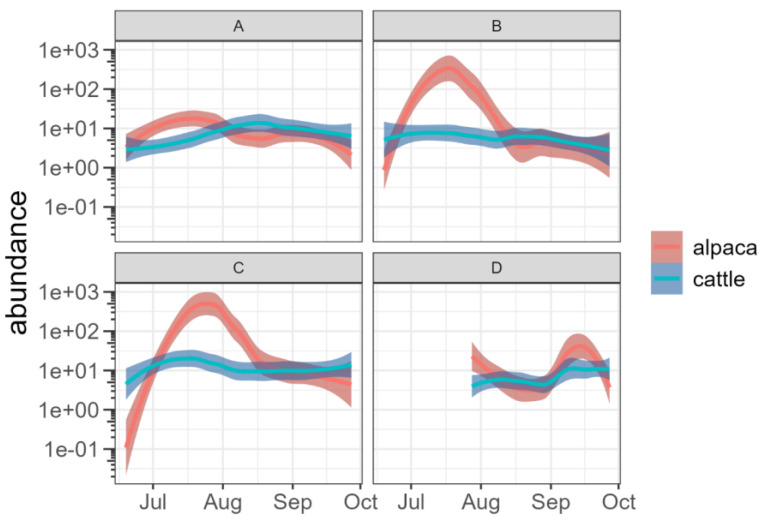
Trends in the number of individuals (abundance) per trap over time at the four pastures (A, B, C and D). In the high-altitude pasture (D) sampling began later than in the others due to the persistence of snow cover. The abundance is reported on a logarithmic scale with base 10.

When considering the trend of species richness and species abundance, the alpaca dung presented higher values of species richness than the cow dung at sites A and B in the month of July (Figure 3) and higher values of species abundance at sites B and C, once again in the month of July (Figure 4).

#### 3.1.4. Trap Ordination

**Figure 5:**
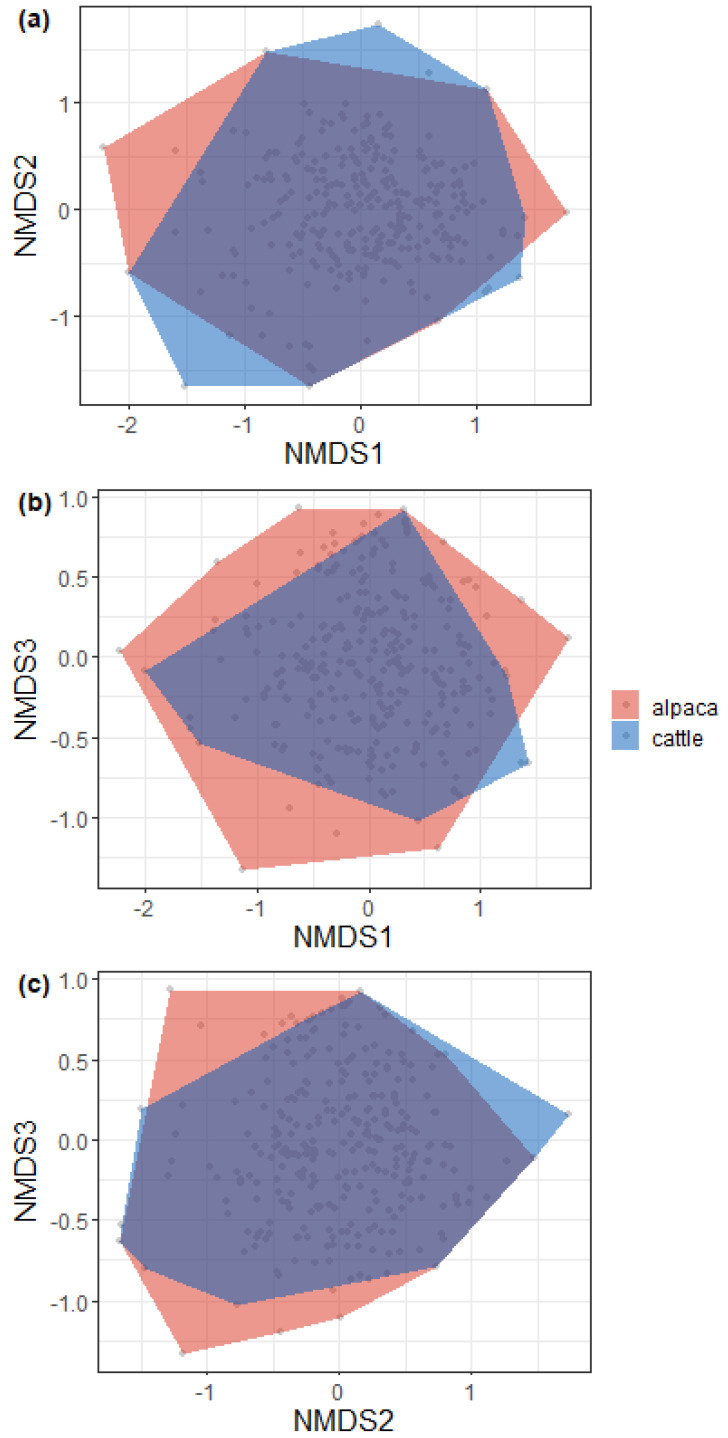
Non-metric multidimensional scaling (nMDS) results ((**a**) = axis 1 vs. axis 2, (**b**) = axis 1 vs. axis 3, (**c**) = axis 2 vs. axis 3). Polygons include all the points of a certain dung type. Each point represents the community of a single trap.

nMDS was performed on three axes with a stress of 0.13. The results show that samples from both treatments overlapped significantly, although alpaca samples were more spread over the multidimensional space, suggesting that the species composition of about fifteen alpaca traps differed from that of all the other cow traps (Figure 5).

Although the two types of dung shared most species, species-specific dung preferences also existed. Based on the fidelity and relative abundance of dung beetles in pitfall traps (IndVal), we found that nine Aphodiinae species were significantly associated with one type of dung. Notably, nearly all these species (8 out of 9) were significantly associated with alpaca dung, whilst only one was associated with cow dung. *E. carinatus* was significantly associated with alpaca dung and showed the highest Indicator Value (Table 2). This result was expected given that this species was overwhelmingly caught in alpaca dung traps (16,984 vs. 329 in cow dung traps). The significant association between alpaca and *L. zenkeri* was also expected given that all 58 individuals collected were retrieved from alpaca dung traps. Neither Scarabeinae nor Geotrupinae species showed a clear preference for one type of dung over the other, although *Onthophagus fracticornis* was found more abundantly in cow dung traps.

### 3.2. Sampling in Dung Pats and Latrines

Dung beetle sampling from the alpaca latrines and cow dung pats present on the pastures confirmed the use of both dung types by dung beetles (Table S1.1 in the Supplementary Materials). We also detected numerous holes in the ground of the latrines.

The attractiveness of the latrines and dung pats to dung beetles changed over time. While the dung pats produced by cows attracted dung beetles for only 3–9 days after being deposited by the animals, the alpaca latrines continued to attract insects for 12–14 days after their creation, i.e., up until their abandonment by the alpacas (Figure S1.1 in the Supplementary Materials). We verified, even if only in anecdotal terms, that the freshness of the pellets laid by the alpaca was, in fact, essential to attract the dung beetles, even outside latrines. In August 2024, four samplings on old latrines which had rarely been used by alpacas over the past 30 days (i.e., they were practically abandoned) led to the collection of only two individuals (one species), while four samplings from small groups of fresh pellets located just outside the latrines resulted in the collection of 105 individuals (10 species).

## 4. Discussion

The field conditions of this study meant that the traps baited with alpaca dung were characterized by the same microclimatic conditions as those baited with cow dung. This was important since microclimatic conditions, particularly temperature (which was near-identical in the two conditions), can influence the local distribution of dung beetles [40]. In consequence, we can attribute the differences in the dung beetle distribution between the two trap types to the attractiveness of the two types of dung to the beetles.

We hypothesized that thousands of years of grazing in the Alps may have triggered a process of adaptation in the local dung beetle communities to the dung produced by the livestock traditionally farmed in the area; this process may have also affected the intestinal microbiota of dung beetles, since the evolutionary history of dung beetles is known to be mirrored in their microbiota [34,35]. Contrary to our expectations, traps baited with cow dung were not found to be more attractive to the local dung beetles than traps baited with alpaca dung. The average number of species per trap did not differ significantly and the nMDS trap ordination for the two treatments largely overlapped, but the total number of individuals and the average number of individuals per trap were both higher in traps baited with alpaca dung. Moreover, abundance and specific richness, which changed with altitude, were both higher in alpaca traps during July at intermediate altitudes. Indeed, IndVal analyses showed that eight out of nine species (all belonging to Aphodiinae) were significantly associated with alpaca traps. The high attractiveness of alpaca traps is in keeping with the results of previous surveys conducted in the United States, where alpacas were first introduced in 1984 [51].

We must acknowledge that the attractiveness of pitfall traps, in which the dung had been manipulated and inserted in fine mesh plastic bags, does not necessarily mean that the dung that is naturally deposited in the pastures is equally attractive and used by dung beetles. The tendency of alpacas to defecate in special latrines was of particular concern from this point of view since we knew that the local dunghills, accumulations of cow dung created by the farmers, were not used by dung beetles at all. The samplings conducted in cow dung pats and alpaca latrines in the second year of this study dispelled any doubts about the dung beetles’ use of latrines. Latrines were found to be used by most of the dung beetle species collected in the pitfall traps located at about the same altitude. Furthermore, the presence of many holes in the ground located underneath the latrines provided strong evidence that tunnelers successfully nested there.

The local alpacas defecate in such a way as to form wide, flat areas of dung (the latrines) that are separated from each other by free ground. It is thought that the animals do this to avoid getting their feet dirty. In fact, the latrines were not configured as accumulations of dung, comparable to a dunghill; instead, they were much thinner, more reminiscent of a high-density crowd of naturally deposited cow dung pats. These considerations may partly explain why alpaca latrines could be safely used by dung beetles.

Samplings of the dung deposited by the ungulates on the pastures confirmed that dung beetles were especially attracted by fresh dung, be it alpaca or cow. The difference was that, while the dung pats produced by cows attracted dung beetles for only a few days after being deposited, the alpaca latrines, being continuously supplemented with new dung, continued to attract them for two consecutive weeks following the latrine’s creation and until its abandonment and consequent desiccation. Naturally, this does not mean that Alpaca latrines support a greater number of dung beetles because cattle defecate every day, meaning that cow dung pats are always present and available, even if their location on the pasture changes.

The ability of dung beetles to locate and exploit a new trophic resource should be discussed considering the availability of dung types, the effects of dung ingestion on the constitution of the beetle’s intestinal microbiome, and the threats derived from the administration of antiparasitic medicines to domestic livestock.

### 4.1. Dung Availability

European dung beetles (i.e., Aphodiinae, Scarabaeinae, Geotrupinae) are known to feed on the dung of all domestic herbivores (sheep, goat, cattle and horse), as well as on human and dog feces. As for the wild fauna, there are many reports indicating that dung beetles make use of dung provided by red deer (*Cervus elaphus* Linnaeus, 1758), fallow deer (*Dama dama* (Linnaeus, 1758)), roe deer (*Capreolus capreolus* (Linnaeus, 1758)), chamois (*Rupicapra rupicapra* (Linnaeus, 1758)), wild boar (*Sus scrofa* Linnaeus, 1758), large rodents such as marmots (*Marmota marmota* (Linnaeus, 1758)), hare (*Lepus* spp.), and rabbits [7,52]. Even though the dung of livestock may attract more individuals than the dung of wild herbivores [11], an exclusive evolutionary link between domestic livestock and dung beetles cannot be assumed. Alpine transhumance itself is not necessarily a determinant of close coevolution between domestic animals and local dung beetle species. Vertical transhumance refers to movements between higher pastures in the summer and lower valleys in the winter. It has been practiced in the Alps since prehistoric times, although livestock are now transported faster and more conveniently using specialized trucks. This type of pastoralism has practical consequences for dung beetles. In fact, a phenological mismatch between the presence of dung beetles and that of the livestock may occur in at least two instances. The arrival and departure periods of the herds (and flocks of sheep or goats) are subject to variability; thus, dung beetles may already be flying over the pastures in late spring, while the herds have yet to arrive, or they may still be active in late autumn, after the herds have left. During these time intervals, dung beetles are forced to look for alternative feeding resources, which will primarily be provided by wild ungulates. The possibility of multiple choices of dung being provided by domestic and wild animals is well exemplified in our study area where, in addition to cattle and alpaca, sheep and goats are also farmed. Moreover, there is no shortage of wild ungulates such as the Alpine ibex (*Capra ibex* Linnaeus, 1758), chamois, and marmot at high altitudes, and wild boar, red deer, and roe deer at low altitudes.

Finally, we must consider that the numbers of both domestic and wild populations of species supplying dung to beetles have undergone many fluctuations over the last centuries, with the Alpine ibex being a prime example. Between the 16th and 18th centuries, the species disappeared from much of its range due to overhunting, with a population of less than 100 individuals remaining in the 19th century, located in and around the Gran Paradiso Mountain massif in Italy. Its recovery to more than 50,000 animals at present is the result of a series of conservation efforts [53,54].

### 4.2. Gut Microbiota and Dung Ingestion

The growth and development of dung beetles, as well as their ecological and evolutionary success, are closely associated with the health of their gut microbiota (which comprise bacteria, archaea, and fungi), the characteristics of which allow them to cope with mammal dung, a nutritionally limited food source [55,56,57,58].

The gut microbiota of dung beetles is remarkably variable among species and individuals [29]. A recent study on three *Onthophagus* species revealed that the composition of the gut microbiota largely depends on the dung ingested and that, despite the taxonomic differences among species, the effective functionality of the microbiota remains relatively stable. If the microbiota lack some microbes, others can compensate, in keeping with the concept of multifunctional redundancy, an intrinsic property of the gut ecosystem [59]. From this perspective, the ingestion of alpaca dung by dung beetles is not a problem, because it would fit into the context of intestinal microbiota variability already widely present in these animals, possibly ensuring increased multifunctional redundancy.

### 4.3. The Use of Antiparasitic Drugs

The use of endectocides (especially ivermectin) in the treatment of domestic livestock over the past decades was recently found to have a negative impact on dung beetle populations and communities around the world [60,61]. Alpine transhumance is a relatively natural livestock management system, and herds are generally not treated with antiparasitic drugs. Alpacas and llamas, collectively referred to as South American Camelids (SACs), are also affected by parasitic infections of the intestinal tract for which they receive specific treatments [62,63]. Thus, we cannot rule out the possibility that the dung beetles using the latrines of these animals may be exposed to these pharmaceuticals. In this context, the fact that these insects may also use the dung produced by wild ungulates is good news as the wild fauna provide a precious reservoir of dung that is free of pharmaceutical contaminants.

Considering the above, the best adaptive survival strategy for alpine dung beetles would have indeed been to develop dung polyphagia combined with notable trophic flexibility. In this context, it is therefore not so surprising that the introduction of an exotic species like the alpaca was well received by the local dung beetle communities.

Alpaca dung is relatively dry and usually deposited in the form of small pellets, although they may sometimes form larger, irregular masses. From this point of view, alpaca dung does not differ very much from that of red, fallow and roe deer. Additionally, the dung’s composition of VOCs changes according to the animal’s diet [12]. Accordingly, if the animals’ diet is the same, one might expect the dung of different ungulates to release volatiles that are similar, at least in part. This may have been the case in the context of the present study since the alpacas and cows were raised in pastures that were largely similar. Under certain circumstances, shifts in resources may also be beneficial for dung beetles in terms of geographic distribution. In Madagascar, for instance, three *Helictopleurus* endemic species shifted to open habitats over the past 1500 years, following the introduction of cattle, and this was accompanied by a rapid expansion of their range [64], possibly mediated by the acquisition of new microbiota.

Finally, considering the results obtained, we can reflect on the possible consequences of an increase in alpaca breeding in the Alps. Although it would require careful monitoring, an increase in the presence of alpacas in the Alpine environment need not provoke too much concern about dung beetle agroecology; on the contrary, it could prove to be naturally sustainable. The attractiveness of alpaca dung to dung beetles and the trophic behavior of these insects toward latrines reduces the need to clean dung from the pastures since the beetles, by feeding on the dung and moving it into tunnels, take care of this work. It is possible that local pastures will even benefit from the transfer of dung-derived nitrogen into the soil, increasing nitrogen uptake by plants and herbage growth, as has already been demonstrated for cow dung [42]. Indeed, McGregor and Brown [65] reported the clear accumulation of nutrients (phosphorus, nitrate–nitrogen, potassium and sulfur) in and around the latrine and suggested that, in the absence of other limiting factors, the transfer of nutrients was sufficient to sustain a high level of grass growth. That said, excessive nutrient transfer could be a cause for concern regarding pasture growth and composition in the long term. The most obvious recommendation for farmers is, therefore, to rotate the pastures dedicated to alpaca grazing. Grazing together with sheep or cattle, as was the case here, may also provide a cost-effective solution to redistributing the local build-up of nutrients [65].

## Figures and Tables

**Table 1:** List of the species collected and their abundances in the traps baited with alpaca or cow dung at the four sampling pastures (A, B, C and D).

		Alpaca					Cow					Tot
Subfamilies	Species	A	B	C	D	Tot	A	B	C	D	Tot	
Geotrupinae	*Anoplotrupes stercorosus* (Hartmann in Scriba, 1791)	0	26	33	12	71	0	5	35	53	93	164
	*Geotrupes spiniger* (Marsham, 1802)	6	7	28	1	42	2	0	21	0	23	65
	*Geotrupes stercorarius* (Linnaeus, 1758)	5	8	7	0	20	4	13	10	0	27	47
Scarabaeinae	*Euoniticellus fulvus* (Goeze, 1777)	2	0	0	0	2	0	0	0	0	0	2
	*Onthophagus fracticornis* (Preyssler, 1790)	127	47	74	6	254	233	60	326	18	637	891
	*Onthophagus joannae* Golijan, 1953	53	5	2	0	60	37	0	0	0	37	97
	*Onthophagus taurus* (Schreiber, 1759)	0	0	0	0	0	0	0	1	0	1	1
Aphodiinae	*Acrossus depressus* (Kugelann, 1792)	16	13	3	0	32	1	1	7	0	9	41
	*Acrossus rufipes* (Linnaeus, 1758)	64	4	13	1	82	4	1	13	0	18	100
	*Agoliinus satyrus* (Reitter, 1892)	0	5	11	72	88	0	0	10	21	31	119
	*Amidorus obscurus* (Fabricius, 1792)	0	3	20	19	42	0	0	82	119	201	243
	*Aphodius pedellus* (De Geer, 1774)	83	36	5	0	124	21	3	1	0	25	149
	*Bodilopsis rufa* (Moll, 1782)	213	272	236	79	800	166	82	156	42	446	1246
	*Chilotorax distinctus* (Müller, 1776)	0	0	0	0	0	1	0	0	0	1	1
	*Colobopterus erraticus* (Linnaeus, 1758)	15	92	31	1	139	49	20	67	0	136	275
	*Esymus pusillus* (Herbst, 1789)	23	56	13	0	92	7	28	46	0	81	173
	*Euheptaulacus carinatus* (Germar, 1824)	37	8128	8478	341	16,984	1	36	281	11	329	17,313
	*Limarus zenkeri* (Germar, 1813)	58	0	0	0	58	0	0	0	0	0	58
	*Oromus alpinus* (Scopoli, 1763)	0	0	1	22	23	0	0	1	18	19	42
	*Otophorus haemorrhoidalis* (Linnaeus, 1758)	0	3	0	0	3	0	0	0	0	0	3
	*Oxyomus sylvestris* (Scopoli, 1763)	7	13	0	4	24	2	0	0	0	2	26
	*Parammoecius corvinus* (Erichson, 1848)	32	4	1	0	37	2	1	0	0	3	40
	*Planolinoides borealis* (Gyllenhal, 1827)	52	3	4	0	59	4	0	6	0	10	69
	*Planolinus fasciatus* (Olivier, 1789)	5	2	66	266	339	2	4	25	76	107	446
	*Rhodaphodius foetens* (Fabricius, 1787)	7	6	2	0	15	16	4	8	0	28	43
	*Teuchestes fossor* (Linnaeus, 1758)	0	4	1	0	5	1	11	4	0	16	21
Tot		805	8737	9029	824	19,395	553	269	1100	358	2280	21,675

**Table 2:** Indicator value analysis of dung beetle preferences for the two dung types (alpaca, cow). IV represents the indicator value and the *p* value represents the significance (* = 0.05, ** = 0.01, *** = 0.001).

Dung Type	Species	IV	*p* Value
alpaca	*Euheptaulacus* *carinatus*	0.654	**
alpaca	*Aphodius pedellus*	0.482	**
alpaca	*Acrossus* *rufipes*	0.325	**
alpaca	*Parammoecius corvinus*	0.299	**
alpaca	*Acrossus* *depressus*	0.275	**
alpaca	*Planolinoides* *borealis*	0.262	*
alpaca	*Oxyomus* *sylvestris*	0.254	**
alpaca	*Limarus zenkeri*	0.243	**
cow	*Amidorus* *obscurus*	0.528	**

## Data Availability

Data are available from the authors upon reasonable request.

## Supplementary Materials

The following supporting information can be downloaded at: https://www.mdpi.com/article/10.3390/insects15120934/s1, Figure S1.1: Differential attractivity of alpaca latrines and cattle dung pats aged 12 days old. Richness and abundance of sample 1 and sample 2 are the mean values of two replicates, while those of sample 3 are based on a single replicate; Table S1:1 List of the species and abundances of dung beetles collected in the alpaca latrines or cow dung pats near pasture A in July and August 2024.
