# Supplementary material for: Thousands of Years of Pastoralism Don’t Count: Coprophagous Beetles Prefer Exotic Alpaca Dung to That of Cattle
Source: Insects. 2024 Nov 27;15(12):934. doi: 10.3390/insects15120934 (PMC11677407; doi:10.3390/insects15120934)
Supplement: Supplementary file 1 [file insects-15-00934-s001.zip › insects-3317309-supplementary.pdf]

SUPPLEMENTARY FILE 1

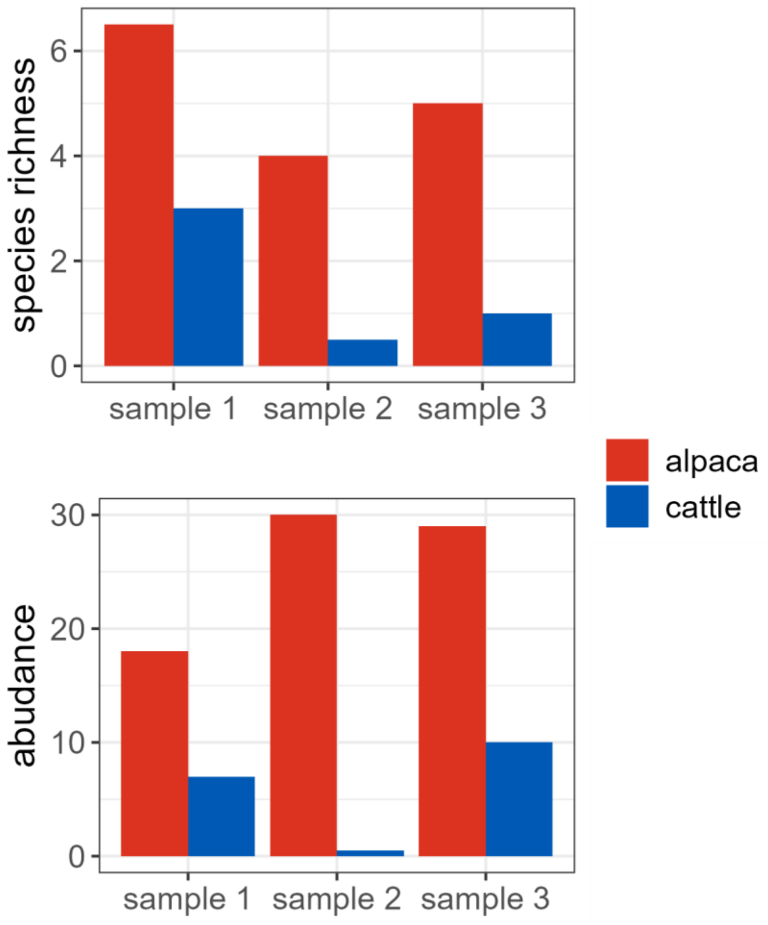

Figure S1. Differential attractivity of alpaca latrines and cattle dung pats aged 12 days old. Richness and abundance of sample 1 and sample 2 are the mean values of two replicates, while those of sample 3 are based on a single replicate.

Table S1. List of the species and abundances of dung beetles collected in the alpaca latrines or cow dung pats near pasture A in July and August 2024.

		alpaca latrine				cow dung pat				Tot
Subfamilies	Species	1	2	3	tot	1	2	3	tot	
Geotrupinae	*Geotrupes spiniger*	0	6	0	6	0	0	0	0	6
	Geotrupes stercorarius	1	1	3	5	0	0	0	0	5
	Trypocopris vernalis	0	0	0	0	5	0	0	5	5
Scarabaeinae	*Euoniticellus fulvus*	1	1	0	2	0	0	0	0	2
	Onthophagus fracticornis	0	21	4	25	0	3	3	6	31
	Onthophagus joannae	8	0	0	8	15	1	1	17	25
	Onthophagus illiricus	0	0	0	0	0	1	0	1	1
	Onthophagus medius	0	0	0	0	1	0	0	1	1
Aphodiinae	*Acrossus depressus*	1	0	0	1	0	0	0	0	1
	Acrossus rufipes	1	0	0	1	0	0	1	1	2
	Aphodius pedellus	5	48	39	92	2	47	73	122	214
	Bodilopsis rufa	10	37	10	57	3	8	14	25	82
	Colobopterus erraticus	2	13	0	15	7	0	23	30	45
	Esymus pusillus	52	6	1	59	4	1	0	5	64
	Otophorus haemorrhoidalis	1	0	1	2	1	0	4	5	7
	Oxyomus sylvestris	10	0	0	10	74	3	0	77	87
	Planolinoides borealis	0	0	0	0	1	0	0	1	1
	Rhodaphodius foetens	2	1	0	3	0	0	1	1	4
	Teuchestes fossor	1	0	0	1	1	1	1	3	4
Tot					287				300	587
